# Development of an Experimental Protocol for Combined Electroporation and Phototherapy Treatment Against Melanoma Cells

**DOI:** 10.3390/ijms27209220

**Published:** 2026-10-17

**Authors:** Enrico Pierluigi Spugnini, Margherita Onofrj, Cosimo Assumma, Maria Condello

**Affiliations:** 1 Biopulse srl, 00144 Rome, Italy; spugnini.vet@gmail.com (E.P.S.); coslab3@gmail.com (C.A.); 2 National Center for Drug Research and Evaluation, National Institute of Health, 01161 Rome, Italy; margheritaonofrj@gmail.com

**Keywords:** melanoma cells, electroporation, acridine orange, phototherapy, programmed cell death

## Abstract

Melanoma is the most common cutaneous tumor with significant challenges for successful pharmacological treatment. The aim of this study was to determine the optimal treatment conditions using a combined therapy based on electroporation and the photoactivation of acridine orange (AO) with a blue LED lamp, designed by Biopulse srl. The effect of combined therapy was investigated by MTT assay and optical microscopy observations. Drug uptake and apoptosis induction were analyzed by flow cytometry. The best treatment schedule was obtained using electric pulses (sequences of eight biphasic pulses lasting 50 + 50 μs with 300 μs interpulse, at a voltage of 300 V), an AO concentration of 0.01 μg/mL, and LED exposure for 30 s. The combined treatment was more effective against melanoma cells than human dermal fibroblast-adult cells. The results demonstrated that electroporation increased AO accumulation, allowing for reduced drug concentrations and exposure times. This combination induced programmed cell death consistent with apoptotic features in melanoma cells, as shown by cell cycle analysis. These preliminary results demonstrated the efficacy of combination therapy based on electroporation and phototherapy with AO on melanoma cells, and they encourage the possibility of testing efficacy on melanoma models in veterinary oncology.

## 1. Introduction

Cutaneous tumors are among the most dangerous and widespread forms of cancer nowadays. Among the diagnosed skin cancers, 20% are cutaneous melanomas. According to the International Agency for Research on Cancer (IARC), projections indicate that 325,000 new cases of melanoma were diagnosed worldwide and 57,000 people died from the disease in 2020 [1]. Furthermore, recent investigations predict that melanoma’s incidence will rise more than 50% from 2020 to 2040 [2]. Treatment for melanoma metastases includes targeted therapy, immunotherapy, and, for unresponsive neoplasms, chemotherapy; meanwhile, electrochemotherapy is confined to the palliation of skin metastases [3,4,5,6]. Chemotherapy agents, currently used as last-line therapy, include dacarbazine, temozolomide, platinum compounds, such as carboplatin, and taxanes, such as paclitaxel [5]. Especially for the treatment of cutaneous metastases, there is a need for a novel second-line therapy where immunotherapy or checkpoint inhibitors fail to achieve local control. A strategy to improve local control of tumors is the combination of some drugs with photoactivation. Photodynamic therapy (PDT) has been investigated for the treatment of a broad spectrum of diseases, primarily for superficial and locally accessible lesions, such as actinic keratosis, Bowen disease, cutaneous squamous cell carcinoma and melanoma [7]. Compared with conventional systemic treatments, PDT offers the potential advantage of localized therapeutic activity, thereby limiting damage to surrounding healthy tissues. The mechanisms of action are now well known, and they include production of reactive oxygen species (ROS), apoptosis induction, and modulation of immune response [8,9]. The biological response depends on photosensitizer chemistry, intracellular localization, light intensity, oxygen availability of tumor cells, and the molecular environment of the target cells. It has been proven that photodynamic or radiodynamic therapy with acridine orange (AO) (referred to as AO-PDT and AO-RDT, respectively) achieved significant specificity and effectiveness both in experimental and clinical tumor settings [10,11,12,13]. AO has been widely used as a photosensitizer, anticancer, and anti-*Plasmodium* drug for malaria. It has a small molecular size and can rapidly enter the target cell and its organelles. When AO is excited by blue light (at approximately 490 nm) or by X-rays, it emits green fluorescence (approximately 525 nm) and generates reactive oxygen species, which promote cell death [14]. This property has been exploited in treating solid tumors by taking advantage of the propensity of AO to bind to acidic milieus, such as those measured within tumors [15].

Finally, among the strategies used to enhance drug uptake by tumor cells, electroporation has gained a lot of interest within the scientific community over the past years due to its high efficiency [16]. Despite the progress in melanoma therapy, there is still a need for effective and well-tolerated therapies for cancer patients. The goal of this study was to assess if the exposure of AO to permeabilizing pulses resulted in increased uptake by targeted cells, and if the subsequent exposure of said cells to exciting blue light resulted in increased effectiveness and promotion of apoptotic cell death.

## 2. Results

### 2.1. The Effects of AO and LED Exposure on Human Melanoma Cells

**Figure 1:**
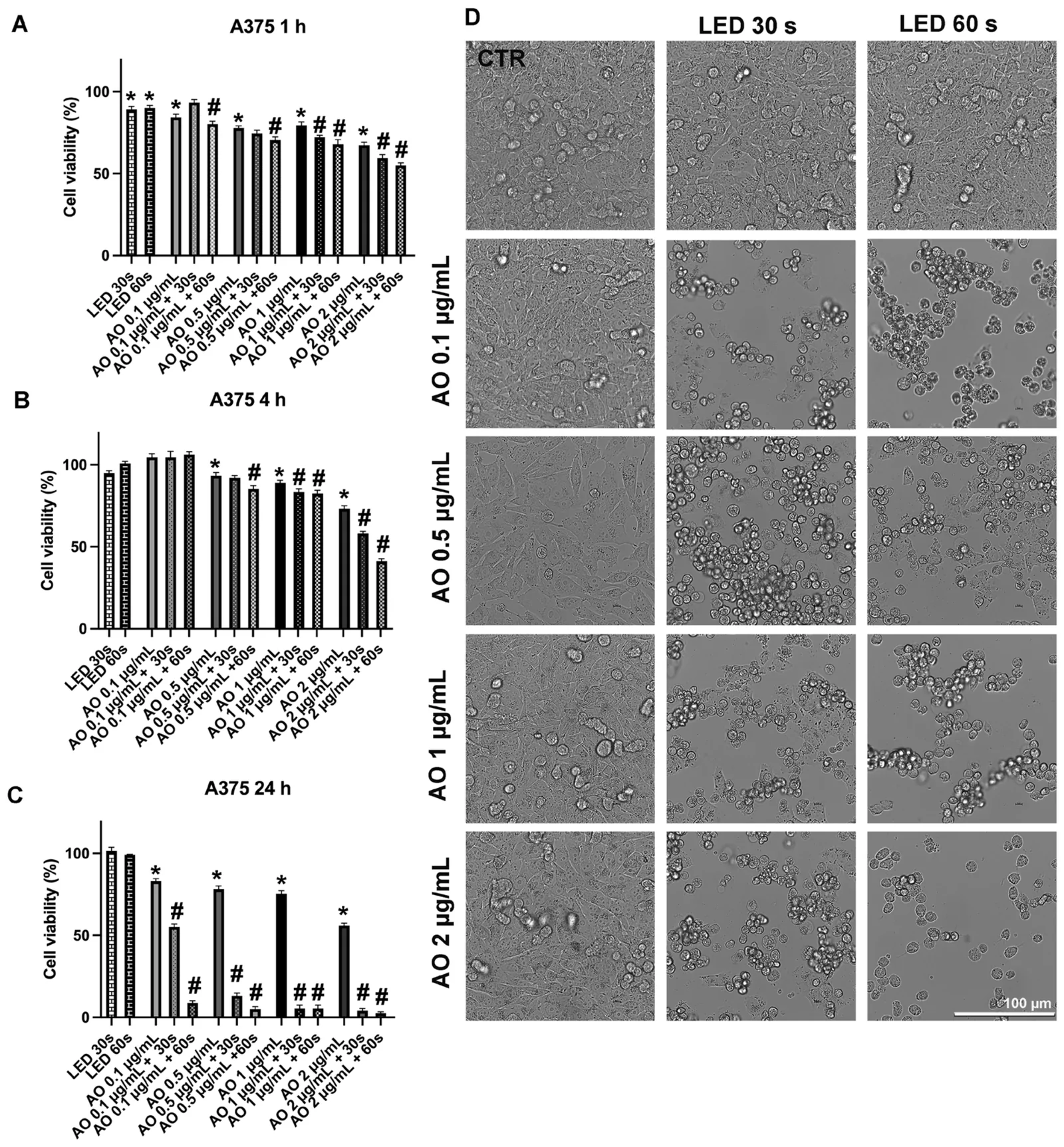
Effects of acridine orange-mediated phototherapy on A375 melanoma cell viability and morphology. Cell viability was evaluated by MTT assay after 1 h (**A**), 4 h (**B**), and 24 h (**C**) of treatment with acridine orange (AO: 0.1, 0.5, 1 and 2 µg/mL) with or without LED exposure for 30 or 60 s. Cell viability is expressed as a percentage relative to untreated control cells (CTR). Data are presented as mean ± SD of three independent experiments. * *p* < 0.05 versus untreated control cells, # *p* < 0.05 versus the corresponding not irradiated AO-treated group. (**D**) Phase contrast microscopy images acquired 24 h after treatment. Cells were exposed to AO (0.1, 0.5, 1, and 2 µg/mL) with or without LED exposure for 30 or 60 s. LED exposure did not affect cell viability and morphology.

To assess the efficacy of AO-PDT on A375 cells, we tested the first range of AO concentrations (0.1–0.5–1.0–2.0 μg/mL) and two LED exposure times (30 and 60 s). Cell viability was evaluated with the MTT assay after 1, 4, and 24 h (Figure 1A–C).

LED exposure for 30 or 60 s induced a slight decrease in cell viability assessed after 1 h, which was recovered after 4 and 24 h. The combination of LED exposure for 30 or 60 s and acridine orange treatment was slightly effective at 0.5, 1 or 2 μg/mL concentrations after 1 h or 4 h (Figure 1A,B). This effect became very strong and evident after 24 h at all the concentrations of AO (Figure 1C).

The observations by phase contrast microscope after 24 h confirmed MTT data (Figure 1D). A375 cells, exposed to the LED lamp for 30 and 60 s, showed the same morphology as untreated cells. After treatment with increasing concentrations of AO, the nuclei of the cells appeared increasingly swollen, even if no signs of cellular suffering were visible. AO activation with an LED lamp for 30 or 60 s induced evident morphological modifications: many cells were detached from the substrate, whereas attached cells had lost their polygonal morphology.

These preliminary results demonstrated the efficacy of LED exposure to activate AO drug on A375 melanoma cells. Then we tested this combination on human dermal fibroblasts to exclude toxic effects on normal cells.

### 2.2. The Effects of AO and LED Exposure on Human Dermal Fibroblasts—Adult (HDF-Ad)

**Figure 2:**
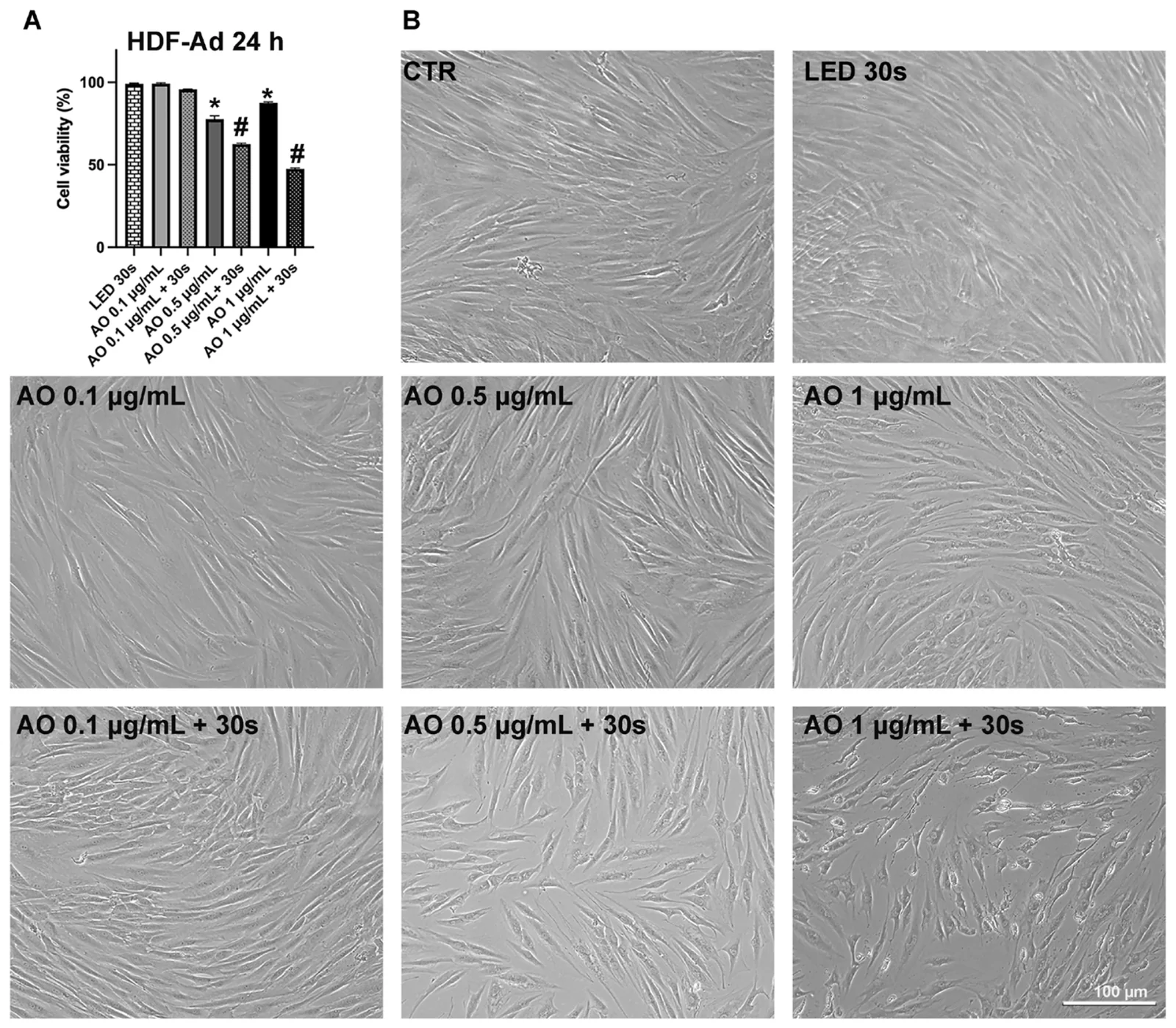
Effects of acridine orange-mediated phototherapy on HDF-Ad cell viability and morphology. (**A**) Cell viability was evaluated by MTT assay after 24 h of treatment with acridine orange (AO: 0.1, 0.5 and 1 µg/mL) with or without LED exposure for 30 s. Cell viability is expressed as a percentage relative to untreated control cells (CTR). Data are presented as mean ± SD of three independent experiments. * *p* < 0.05 versus untreated control cells, # *p* < 0.05 versus the corresponding not irradiated AO-treated group. (**B**) Phase contrast microscopy images acquired 24 h after treatment. Cells were exposed to AO (0.1, 0.5 and 1 μg/mL) with or without irradiation for 30 s LED exposure did not affect cell viability and morphology.

The effects of AO and LED exposure on the viability of human dermal fibroblasts-adult (HDF-Ad) were assessed using the MTT assay (Figure 2A) 24 h after treatment.

The exposure to LED alone for 30 s did not cause significant changes in cell viability compared to the control. Treatment with AO at a concentration of 0.1 μg/mL, both with or without LED exposure, did not induce a significant reduction in cell viability, showing values comparable to those of the control. When cells were treated with AO at 0.5 μg/mL, a moderate reduction in cell viability was observed, which was more pronounced following the combination with exposure to LED light. At the highest concentration (1 μg/mL), HDF-Ad cells treated with AO in the absence of light showed viability values like those observed after treatment with AO 0.5 μg/mL, while the association with LED exposure determined a marked reduction in cell viability (about 50%).

The effect on cell viability was confirmed by optical microscopy observations (Figure 2B). HDF-Ad exposed to LED for 30 s showed a morphology like the control cells, appearing adherent to the substrate and taking on a narrow, elongated shape. This morphology was also observed following treatment with 0.1 μg/mL AO, both in the absence and presence of light, as well as after treatment with 0.5 μg/mL AO or 1 μg/mL AO. Only some nuclei of cells treated with 1 μg/mL appeared swollen compared to untreated cells or other samples treated with lower AO concentrations. When the fibroblasts were treated with 0.5 μg/mL AO and exposed to an LED lamp, a decrease in cell density and a slight alteration in morphology were observed. The morphological change and reduction in cell number were even more evident following treatment with 1 μg/mL AO and light exposure, where cells had lost adhesion to the substrate and assumed a rounder morphology compared to those treated with 1 μg/mL AO alone. Overall, these results demonstrated a light effect of higher concentrations of AO photoactivation on human dermal fibroblasts.

### 2.3. The Efficacy of Electroporation on A375 Melanoma Cells Treated with AO and LED Exposure

We continued our studies to find the best conditions of AO photoactivation for melanoma treatment without toxic effects on normal cells. To limit these toxic effects, it would have been necessary to reduce the concentration of the drug, but this would have limited the effect on melanoma cells. For this reason, we chose to sensitize melanoma cells to AO by electroporation.

**Figure 3:**
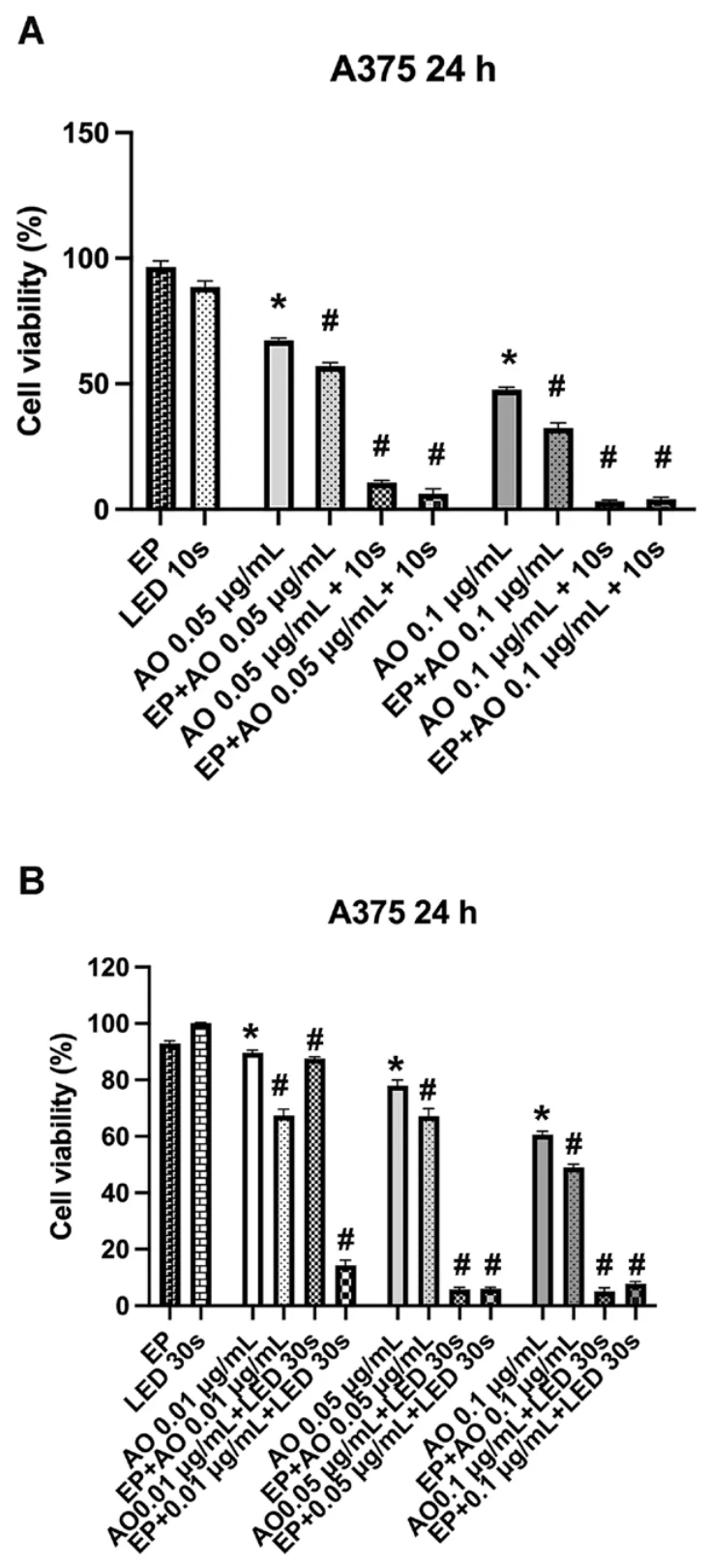
Effects of acridine orange-mediated phototherapy combined with electroporation on A375 melanoma cell viability. (**A**) Cell viability was evaluated by MTT assay 24 h after treatment with acridine orange (AO: 0.05 and 0.1 µg/mL), electroporation (EP), or their combination, with or without LED exposure for 10 s. (**B**) Cell viability was evaluated by MTT assay 24 h after treatment with acridine orange (AO: 0.01, 0.05 and 0.1 µg/mL), electroporation (EP), or their combination, in the absence or presence of LED exposure for 30 s. Cell viability is expressed as a percentage relative to untreated control cells (CTR). Data are presented as mean ± SD of three independent experiments. * *p* < 0.05 versus untreated control cells, # *p* < 0.05 versus the corresponding non-irradiated AO-treated group.

We used two different treatment modalities: we maintained high AO concentrations (0.05 and 1 μg/mL) while decreasing the light exposure time (10 s) (Figure 3A). Conversely, we also used a lower concentration (0.01, in addition to 0.05 and 1 μg/mL) while maintaining the exposure time at 30 s (Figure 3B). In both cases, we evaluated the effect on cell viability by MTT assay after electroporation.

As shown in Figure 3A, the application of electroporation reduced the viability of A375 cells exposed to both AO concentrations (0.05 and 1 μg/mL). The effect was more evident when A375 cells were also exposed to an LED lamp for 10 s, where cell viability was about 5% for both concentrations.

Quantitative MTT data were confirmed by optical microscopy observations (Figure S1). When A375 cells were electroporated and then treated with AO (0.05 or 0.1 μg/mL), we observed many rounded cells in comparison with control cells or cells treated with AO alone (0.05 or 0.1 μg/mL). Moreover, all A375 cells, after electroporation, treatment with AO solution, and finally exposure to LED, were rounded and died.

A similar effect was observed in the other treatment modality (Figure 3B). In particular, the use of a low concentration of acridine orange highlighted how electroporation played an important role in enhancing the effect of photoactivation. After electroporation of AO 0.01 μg/mL-treated cells, the viability was about 65%, and it decreased by approximately 20% compared to cells treated alone. The photoactivation of cells treated with AO 0.01 μg/mL for 30 s decreased viability only slightly compared to the non-photoactivated sample. When A375 cells were electroporated, treated with AO 0.01 μg/mL, and then exposed to LED for 30 s, cell viability was about 14%. The images from optical microscopy confirmed the MTT data, and they clearly showed that the triple combination (electroporation, treatment and photoactivation) induced noticeable morphological changes (Figure S2). In particular, the concentration of AO 0.01 μg/mL, and the LED exposure for 30 s were the best conditions of treatment to combine with the electroporation.

### 2.4. The Effect of Electroporation on AO Intracellular Accumulation

Taking advantage of the intrinsic autofluorescence properties of AO (green emission at about 525 nm and red emission at 575 nm), we evaluated by flow cytometry whether the adjuvant effect of electroporation was due to increased intracellular accumulation of AO. The A375 cells were electroporated and subsequently treated with different concentrations of AO (0.01, 0.05, 0.1, 0.5 µg/mL) for 30 min or 3 h and analyzed by flow cytometry.

**Figure 4:**
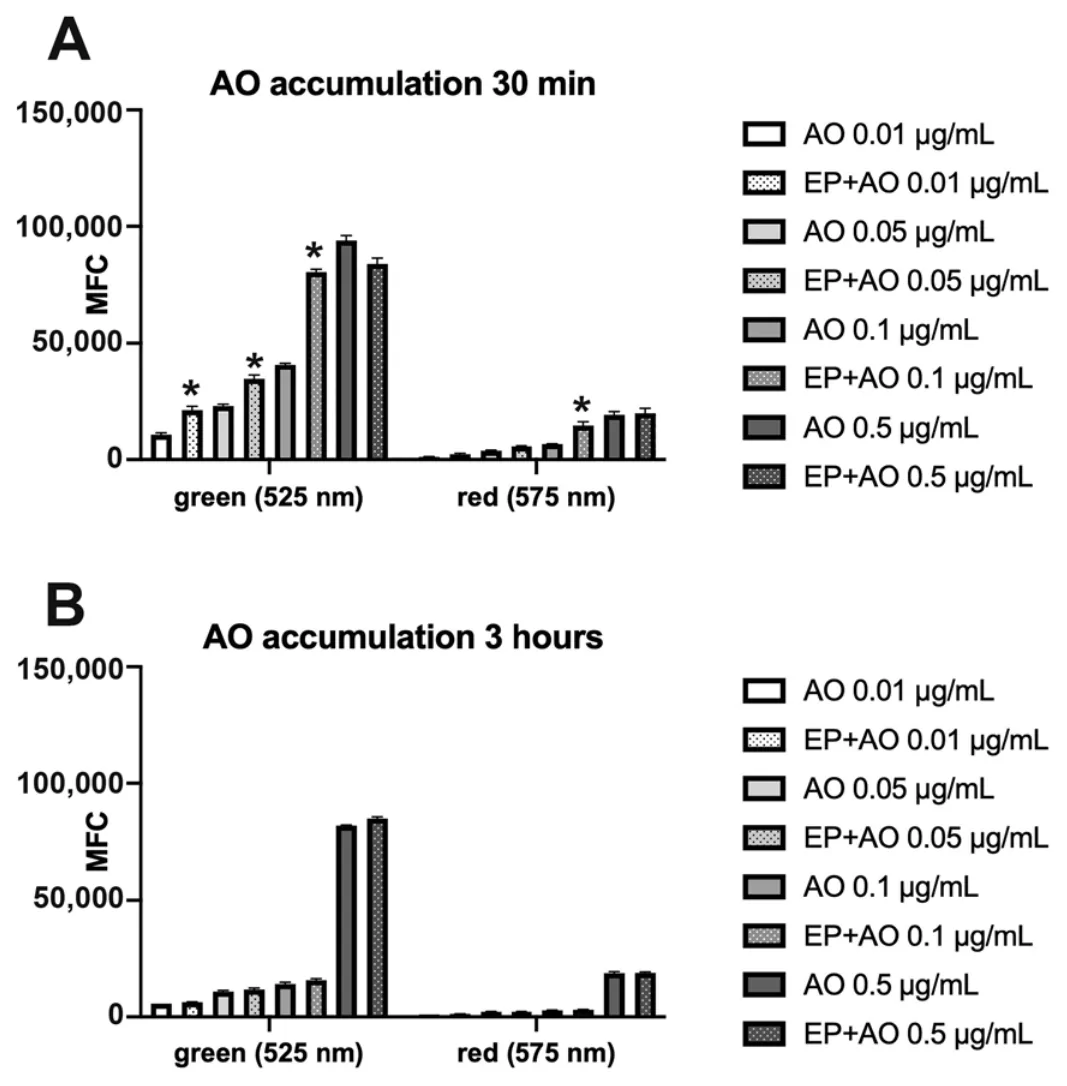
Flow cytometric analysis of intracellular accumulation of acridine orange after electroporation on A375 melanoma cells. (**A**,**B**) Intracellular acridine orange (AO) accumulation following incubation with AO (0.01, 0.05, 0.1 and 0.5 µg/mL) with or without electroporation (EP). Mean fluorescence channel (MFC) was measured in the green (525 nm) and red (575 nm) channels after 30 min (**A**) and 3 h (**B**) of incubation. Data are expressed as mean ± SD of three experiments. * *p* < 0.05 versus the corresponding AO-treated non-electroporated group.

As shown in Figure 4A, after 30 min of treatment with 0.01, 0.05 and 0.1 μg/mL AO of electroporated A375 cells, an increase in green fluorescent signal was recorded compared to non-electroporated cells. This effect occurred only at short times (30 min), as it stabilized after just three hours at all concentrations and for both fluorescence channels (Figure 4B). These analyses suggested that electroporation increased drug retention inside melanoma cells and consequently increased the toxic effect of photo-activated AO, especially at low concentrations.

### 2.5. The Effect of the Combined Treatments on Cell Cycle Distribution

**Figure 5:**
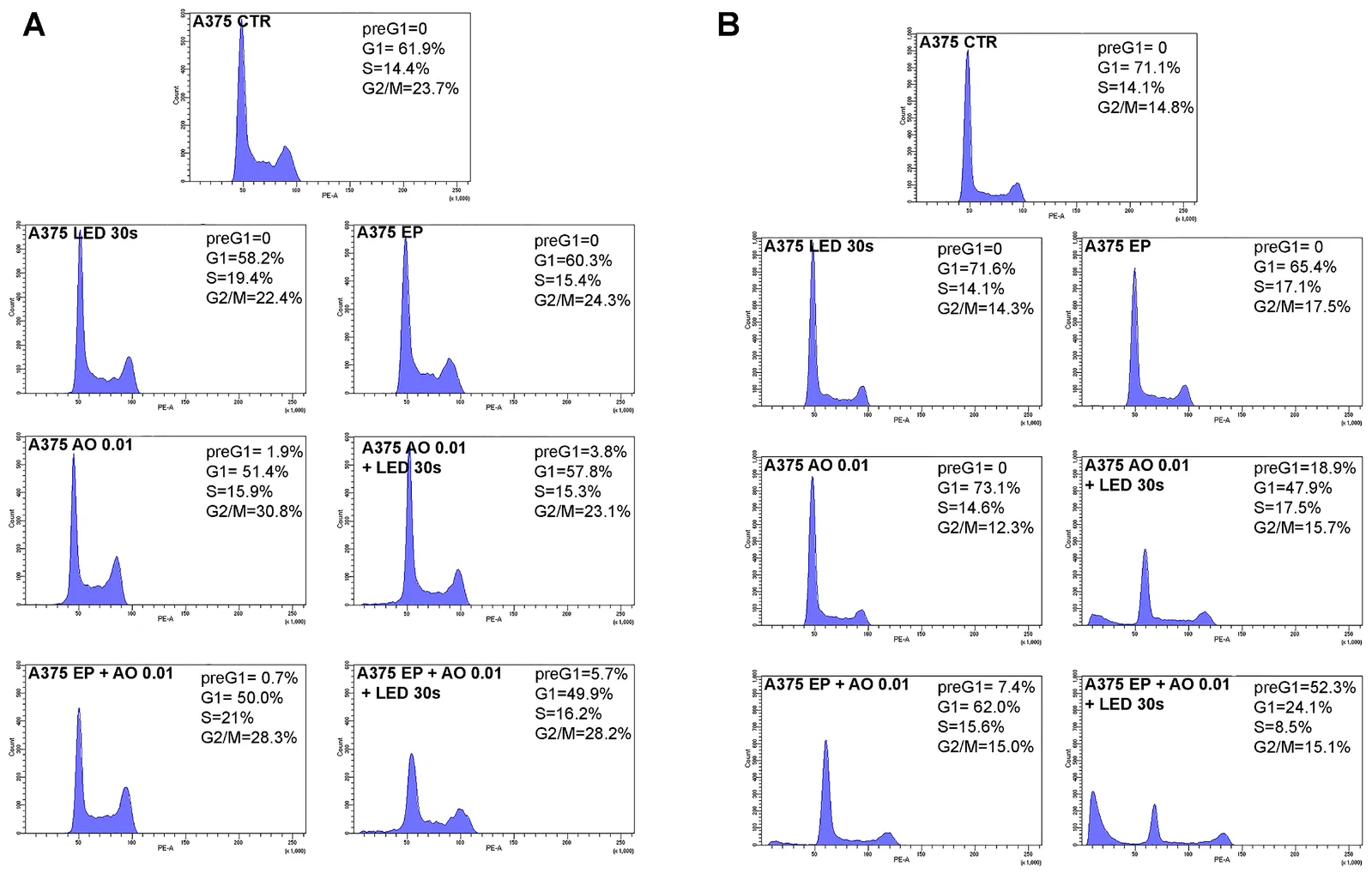
Flow cytometric analysis of cell cycle perturbation induced by acridine orange-mediated photodynamic treatment and electroporation in A375 melanoma cells. (**A**,**B**) Analysis performed 24 h (**A**) and 48 h (**B**) after treatment with acridine orange (AO; 0.01 µg/mL), electroporation (EP), and LED exposure (30 s), alone or in combination. Representative histograms of DNA content showed the percentage of cells in the pre-G1, G1, S, and G2/M phases. Untreated cells (CTR) are included for comparison. The appearance of a pre-G1 population indicates DNA fragmentation and was used as a marker of treatment-induced cell death.

To understand the cell death mechanism involved, we performed cell cycle analysis after 24 and 48 h of treatment by DNA staining of samples, which showed a significant reduction in the MTT assay (Figure 5). The analysis at 24 h did not show significant alterations in cell cycle profiles (Figure 5A). After 48 h (Figure 5B), the cell cycle profiles of samples subjected to electroporation or illumination alone did not show significant alterations in the different phases of the cell cycle compared to untreated cells. When A375 cells were treated with 0.01 μg/mL AO and then exposed to an LED lamp for 30 s, we observed the appearance of a preG1 peak (18.9%), confirming AO activation. This phase noticeably increased when A375 cells were electroporated before treatment and exposure to LED (about 52.3%) compared to other samples. However, no alterations in other phases, such as S and G2/M, were observed.

These results suggested the involvement of programmed cell death consistent with apoptotic features when melanoma cells were electroporated and then illuminated for AO activation.

## 3. Discussion

Dynamic phototherapy involves the use of photosensitizing molecules as chemotherapeutic agents and their activation through exposure to visible light [17]. The use of AO as a photosensitizer is particularly advantageous, as it is a compound characterized by moderate systemic toxicity and the ability to selectively target tumor cells [16]. AO-PDT is widely used in the treatment of skin tumors, including melanoma, thanks to the numerous advantages that this therapeutic strategy offers. In particular, skin tumors present favorable characteristics for the application of the therapy, as they are located on the body surface and therefore easily accessible to light irradiation. The drug’s dependence on light activation gives the treatment a high spatial and temporal selectivity, allowing the cytotoxic action to be limited to the target areas and reducing damage to surrounding healthy tissue. This represents a significant advantage over other therapeutic protocols, such as radiotherapy, chemotherapy, and extensive surgical excision, which may be associated with greater side effects [17,18].

In a preliminary phase, we evaluated the cytotoxic effect of phototherapy on A375 cells treated with different concentrations of AO (0.1 μg/mL, 0.5 μg/mL, 1 μg/mL, 2 μg/mL) and exposure times to blue light (30 and 60 s) by MTT assay and microscopic observations (Figure 1). The results, analyzed at 1, 4, and 24 h after treatment, highlighted a moderate effect on viability at short times (1 and 4 h), while at 24 h a remarkable reduction in cell viability was observed, above all following photoactivation of the drug with an LED lamp. The data therefore showed that the effect of AO was dose- and time-dependent. Based on these results, AO concentrations (below 1 μg/mL) and lamp exposure times (below 30 s) were chosen for future experiments.

The treatment was subsequently evaluated on HDF-Ad to verify the possible appearance of undesirable effects on healthy cells (Figure 2). HDF-Ad were treated with AO at concentrations of 0.1 μg/mL, 0.5 μg/mL, and 1 μg/mL, with and without light exposure for 30 s. The data demonstrated a toxic effect, especially at the highest concentration (1 μg/mL), but of less intensity than that induced in A375 cells, suggesting a greater sensitivity of melanoma cells to treatment. This selectivity could be attributed to the characteristics of tumor cells, such as the more acidic intracellular environment and the higher nucleic acid content, conditions that favor the intracellular accumulation of AO [19].

Our previous studies have shown that electroporation can enhance the accumulation and the efficacy of chemotherapeutic agents, such as Doxorubicin and Mitomycin C [20]. Based on these studies, the use of electroporation has been hypothesized to increase the uptake and efficacy of AO, thus exploiting its effects to reduce drug concentrations and/or LED exposure times. So, we tested two different treatment modalities: we maintained high AO concentrations (0.05 and 0.1 μg/mL) while decreasing the light exposure time (10 s). Conversely, we also used a lower concentration (0.01, in addition to 0.05 and 0.1 μg/mL) while maintaining the exposure time at 30 s. The results showed that a shorter exposure time (10 s) was still sufficient to induce a marked reduction in cell viability (Figure 3A). From a clinical perspective, the possibility of reducing the exposure time to light could contribute to reducing the duration of the therapeutic procedure, improving the overall tolerability of the treatment.

To highlight more clearly the contribution of electroporation, a lower concentration of AO, equal to 0.01 μg/mL, was tested with 30 s of LED exposure time (Figure 3B). Under these conditions, the reduction in cell viability was significantly greater in the combined treatment with electroporation, compared to treatment with AO and light alone, an aspect of potential clinical interest for the reduction in drug side effects.

The images obtained by phase-contrast optical microscope confirmed MTT data, highlighting morphological alterations compatible with cell death, with a clearly dose- and light-dependent effect.

Preliminary observations by optical microscopy were confirmed by flow cytometric analysis of AO accumulation (Figure 4). AO concentrations (0.01, 0.05, 0.1, and 0.5 μg/mL, with or without electroporation) were selected based on preliminary experiments; higher doses produced off-scale fluorescence signals, preventing reliable analysis. After 30 min of treatment (Figure 4A), the data show a significant increase in AO accumulation on the electroporated and treated samples compared to those treated with the drug alone, especially in the green fluorescence channel, suggesting nuclear localization. Conversely, 3 h after treatment (Figure 4B), mean fluorescence was comparable between electroporated/treated cells and cells treated without electroporation, suggesting a transient membrane permeabilization mediated by a type of reversible electroporation [21]. Indeed, following the application of electrical pulses, the formation of transient pores in the plasma membrane improved drug intracellular uptake, achieving the nuclear target. Subsequently, the restoration of membrane integrity and the activation of regulatory mechanisms may contribute to the reduction in the drug cytoplasmic concentration.

To further investigate the cell death mechanism, the cell cycle was analyzed by flow cytometry after 24 and 48 h of treatment (Figure 5). The cells were treated with 0.01 μg/mL AO, with or without LED exposure and/or electroporation. This concentration was selected based on previous MTT results, as it allows us to appreciate the contribution of electroporation to the reduction in cell viability induced by the photoactivated drug. After 24 h, we did not observe any significant difference. At 48 h post-treatment, the increase in the pre-G1 hypodiploid population, resulting from DNA fragmentation, was more marked in the AO-treated samples, both in the presence and absence of light. This increase was particularly evident after the triple combination (EP + AO + light), clearly indicating the activation of programmed cell death consistent with apoptotic features.

These data did not fully align with the results of the MTT assay, which observed a marked reduction in cell viability already 24 h after treatment. However, the two analyses study two different biological end-points: the MTT assay measures mitochondrial damage and the consequent reduction in metabolic activity, while the cell cycle analysis highlights DNA alterations. AO, once activated by light exposure, induces the production of reactive oxygen species (ROS) within the cell [15]. The increase in these species determined a state of oxidative stress that initially compromised mitochondrial function. Damaged mitochondria, in turn, amplify the production of ROS and release pro-apoptotic proteins, triggering the programmed cell death cascade [22]. The temporal progression of these biological events may explain the different timing observed between the reduction in metabolic viability and the evident appearance of the apoptotic fraction in the cell cycle. In conclusion, programmed cell death consistent with apoptotic features was due both to direct action on DNA, as shown by optical observations and flow cytometric analysis of AO uptake, and to amplified oxidative stress, as documented by literature data.

In conclusion, this study demonstrated that the combination of electroporation and phototherapy with AO was a promising strategy for melanoma treatment, capable of enhancing therapeutic efficacy while simultaneously reducing systemic toxicity. These results, obtained on a 2D model of melanoma, will be confirmed on a 3D multicellular model composed of A375 cells, HDF-Ad, and keratinocytes (HaCaT), fine-tuned by our group [23]. These in vitro data will offer the possibility of initiating veterinary oncology studies using animal models of metastatic melanoma.

## 4. Materials and Methods

### 4.1. Cell Cultures

Malignant human melanoma cell line (A375) was purchased from American Type Culture Collection (CRL-1619, ATCC, Manassas, VA, USA). A375 cell line was grown in RPMI 1640 medium (Euroclone, Carmlington, UK) supplemented with 10% FBS (Hyclone Laboratories, Logan, UT, USA), L-glutamine (2 mM) (Euroclone), and 1% penicillin (50 U/mL)–streptomycin (50 µg/mL) (Euroclone). We used A375 cells for experiments starting from culture passage number 4.

Human dermal fibroblast—adult (HDF-Ad) cells were purchased from Cytion (300606, Cytion, Heidelberg, Germany). They grew in MEM medium (Euroclone) with 10% FBS, human recombinant FGF2 2 ng/mL (Applied Biological Materials Inc., Richmond, BC, Canada), 1% non-essential amino acids, 1% L-glutamine, and 1% penicillin (50 U/mL)–streptomycin (50 µg/mL). We used HDF-Ad cells for experiments starting from culture passage number 2.

All of them grew in a humidified atmosphere at 37 °C, 5% CO_2_. Mycoplasma testing was routinely performed with the Hoechst33258 (Abcam, Cambridge, UK) staining procedure [24].

### 4.2. Electroporation

Trains of biphasic electric pulses were generated with a modified clinical electroporator certified for veterinary use and adapted for laboratory applications (Onkodisruptor^®^-Lab, Biopulse Biotech, Rome, Italy). Cells were suspended and placed in electroporation cuvettes with a 4 mm electrode gap, and exposed to sequences of eight biphasic pulses lasting 50 + 50 μs with 300 μs interpulse, at a voltage of 300 V (nominal field strength 750 V/cm; 3.2 ms total time).

### 4.3. Cell Treatments

The experiments were performed by treating A375 cells with different concentrations of AO (0.01, 0.05, 0.1, 0.5, 1 and 2 µg/mL) for 30 min. After washing with medium, cells were exposed to blue light for 10, 30 or 60 s and then incubated for up to 24 h.

The electroporation pulse was applied before treatment with AO.

### 4.4. Home-Made Device for Blue Light Sources

A lamp designed and built to the following specifications was used: six 3W blue LEDs—each with a peak wavelength of 460 nm, a luminous flux of 55 lumens, and a 110° beam angle—were arranged and soldered onto a circuit board approximately 7 cm long to ensure full coverage of the illumination area. The LEDs were housed in a custom-made enclosure measuring 16 × 16 cm with a height of approximately 8 cm. The board was powered via an externally mounted converter; the issue of heat generation from the LEDs (which was nonetheless lower than that of traditional mercury-vapor lamps) was addressed by attaching an aluminum heat sink directly behind the LEDs, paired with a 120 mm axial cooling fan mounted externally near the circuit board. Additionally, ventilation slots were incorporated into the bottom of the enclosure to facilitate airflow.

During exposure, the lamp box is placed over the plate, which rests on the work surface of the hood with its lid on. The LEDs are 7.8 cm above the cells. The inner walls of the box are reflective. The irradiance at the center of the plate, calculated from the rated output of the LEDs and the geometry of the box, is 50 ± 15 mW/cm^2^. The light dose is therefore 0.5, 1.5 and 3 J/cm^2^ for 10, 30 and 60 s, with the same relative uncertainty.

### 4.5. MTT Assay

Cell viability was assessed using the MTT assay (Sigma Aldrich, St. Louis, MO, USA) to evaluate the effects of electroporation, AO and blue light exposure on A375 melanoma cells (after 1, 4 or 24 h) and on human dermal fibroblast-adult (after 24 h). At the end of different treatment conditions, cell medium was removed; the samples were washed with phosphate-buffered saline (PBS, Sigma Aldrich) and incubated with 1 mg/mL MTT solution for 2 h at 37 °C. After discarding the MTT solution, the samples were lysed with dimethyl sulfoxide (DMSO, Sigma Aldrich), and absorbance was measured at 570 nm using a Varioskan Lux multimode microplate reader (Termofisher Scientific, Waltham, MA, USA). The optical density values of the different wells were used to express the percentage of viable cells, calculated as follows: (mean absorbance of treated sample/mean absorbance of the control sample) × 100.

### 4.6. Phase Contrast Microscopy

A phase contrast optical microscope (ECLIPSE Ti2 light microscope, Nikon Europe, Amsterdam, The Netherlands) connected to a digital camera was used to observe the effect of the different treatments on the morphology of melanoma or human dermal fibroblast-adult cells after treatments. Images were subsequently processed using the Adobe Photoshop 2026 program.

### 4.7. Drug Uptake

To assess whether electroporation enhanced drug uptake, AO accumulation was analyzed by flow cytometry after 30 min and 3 h of treatment with AO (0.01–0.05–0.1 and 0.5 µg/mL) in A375 melanoma cells, with and without electroporation. At the end of the treatment, samples were centrifuged, washed with PBS, and analyzed for AO content evaluation. Fluorescence intensity was measured using a BD LSR II flow cytometer (Becton Dickinson, Franklin Lakes, NJ, USA) equipped with a 15 mW, 488 nm, air-cooled argon ion laser. Fluorescence emissions were collected through 530 nm and 575 nm band-pass filters and acquired in log mode. AO content was expressed as mean fluorescence channel (MFC) calculated using the FACSDiva Software 8.0.1.

### 4.8. Cell Cycle Analysis

The effects of treatments on the cell cycle were analyzed by flow cytometry 24 h and 48 h after treatments, for longer durations compared to MTT assays, since DNA damage is a later event than mitochondrial damage. The culture medium was removed and used to resuspend the cells detached with EDTA and trypsin. Cell suspensions were washed with PBS, fixed in 70% ethanol, and stored at 4 °C for 48 h. Following a PBS wash, samples were incubated with RNase (0.1 mg/mL) and propidium iodide (40 µg/mL) in PBS. Cell cycle distribution was determined using a BD LSR II flow cytometer (Becton Dickinson, Franklin Lakes, NJ, USA) equipped with a 15 mW, 488 nm, and air-cooled argon ion laser. Fluorescence emissions were collected through a 575 nm band-pass filter and acquired in linear mode. Data were analyzed with the FACS Diva software 8.0.1.

### 4.9. Statistical Analysis

The results obtained from three independent experiments were expressed as mean ± standard deviation. One-way analysis of variance (ANOVA) and Bonferroni and Dunnett post hoc analyses were performed to identify differences among all samples using GraphPad Prism 10 Software (GraphPad, San Diego, CA, USA). The level of significance was set at *p* < 0.05.

## Data Availability

Dataset available on request from the authors.

## Supplementary Materials

The following supporting information can be downloaded at: https://www.mdpi.com/article/10.3390/ijms27209220/s1.

## Abbreviations

The following abbreviations are used in this manuscript:

IARC	International Agency for Research on Cancer
AO	Acridine orange
AO-PDT	Acridine orange photodynamic therapy
AO-RDT	Acridine orange radiodynamic therapy
LED	Light-emitting diode
CTR	Control
EP	Electroporation
HDF-Ad	Human dermal fibroblasts—adult
MFC	Mean fluorescence channel
ROS	Reactive oxygen species
ATCC	American Type Culture Collection
PBS	Phosphate-buffered saline
DMSO	Dimethyl sulfoxide
ANOVA	One-way analysis of variance
