# Supplementary material for: Development of an Experimental Protocol for Combined Electroporation and Phototherapy Treatment Against Melanoma Cells
Source: Int J Mol Sci. 2026 Oct 17;27(20):9220. doi: 10.3390/ijms27209220 (PMC13705307; doi:10.3390/ijms27209220)
Supplement: Supplementary file 1 [file ijms-27-09220-s001.zip › ijms-4576039-supplementary.pdf]

Development of an experimental protocol for combined electroporation and phototherapy treatment against melanoma cells

Enrico Pierluigi Spugnini [1], Margherita Onofrj [2], Cosimo Assumma [1], and Maria Condello [2,*]

Supplementary Figure S1

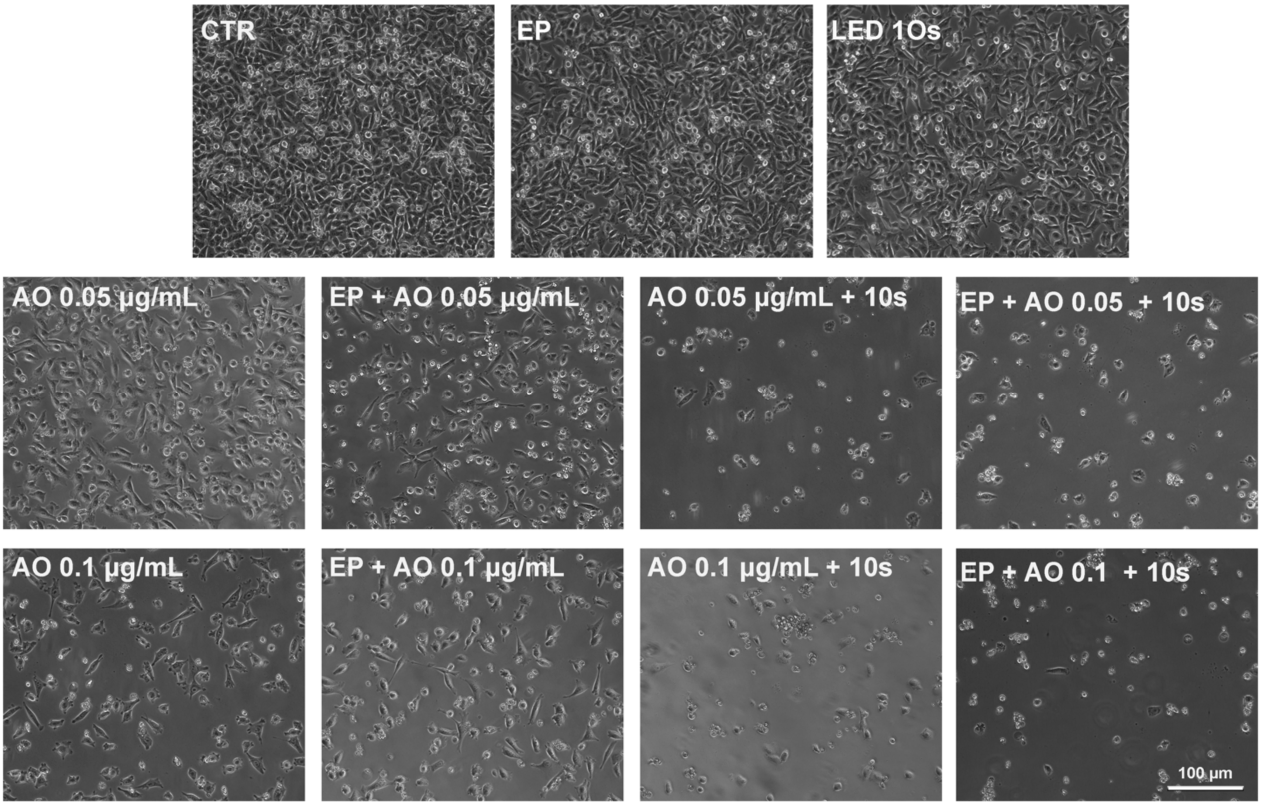

Phase contrast microscopy images acquired 24 h after treatment. Cells were exposed to AO (0.05 and 0.1 µg/mL), EP, or their combination with or without 10 s of LED exposure.

Supplementary Figure S2

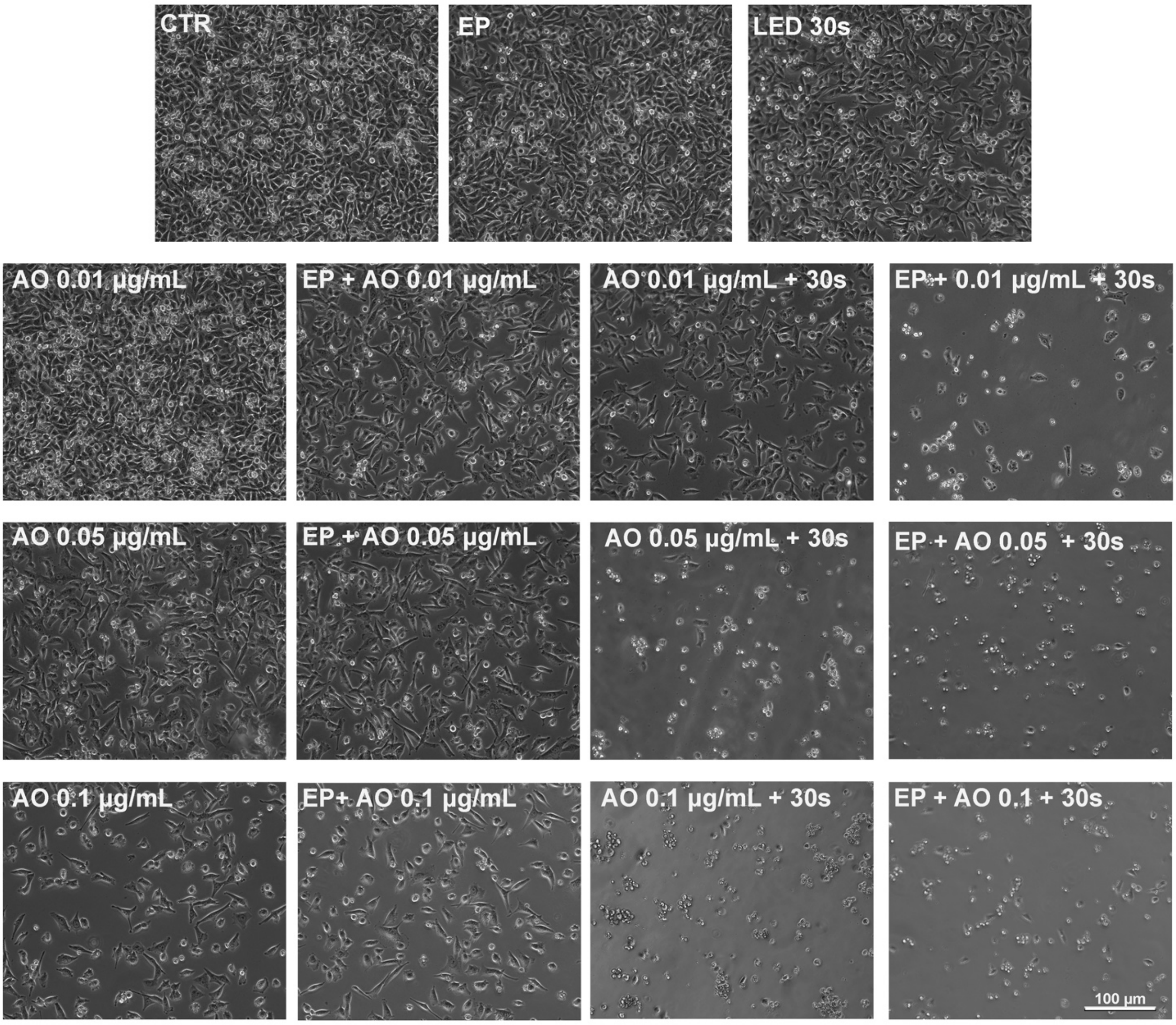

Cells were exposed to AO (0.01, 0.05 and 0.1 µg/mL), EP, or their combination with or without 30 s of LED exposure.
